# C-reactive protein-guided antibiotic therapy and prognostic value in acute infective exacerbation of COPD

**DOI:** 10.6026/973206300222240

**Published:** 2026-04-30

**Authors:** Sreeuthra Baskaran, Gnanaguru S

**Affiliations:** 1 Department of Acute Medicine, Mid and South Essex Trust, UK; 2 Department of General Medicine, Government Thoothukudi Medical College, Tamil Nadu, India

**Keywords:** Chronic obstructive pulmonary disease (COPD), C-reactive protein (CRP), antibiotic stewardship, exacerbation, prognostic marker

## Abstract

Acute exacerbations of chronic obstructive pulmonary disease (COPD) frequently result in excessive empirical antibiotic use despite heterogeneous infectious triggers. Therefore, it is of interest to evaluate C-reactive protein (CRP) -guided antibiotic therapy and its prognostic significance in 50 hospitalized patients with acute infective exacerbation. Antibiotics were initiated when CRP ≥50 mg/L, while lower values were managed conservatively unless deterioration occurred. Patients with elevated CRP demonstrated greater initial inflammatory and clinical severity, whereas those with lower CRP improved without antibiotics and without adverse outcomes. CRP-guided management reduced unnecessary antibiotic exposure and supported its utility as a biomarker for therapeutic decision-making and short-term prognosis.

## Background:

Chronic obstructive pulmonary disease (COPD) is a progressive inflammatory airway disorder and remains one of the leading causes of global mortality and disability. Acute exacerbations of COPD (AECOPD) represent critical clinical events characterized by worsening dyspnoea, increased sputum production and heightened airway inflammation [1]. These episodes accelerate lung function decline, impair quality of life and substantially increase hospitalization rates and healthcare expenditure. Frequent exacerbations are also associated with higher long-term mortality [2]. The etiology of AECOPD is heterogeneous. Bacterial infections account for approximately 40-50% of cases, while viral infections, environmental pollutants and host inflammatory responses contribute significantly to the remainder [3]. Despite this variability, antibiotic therapy is frequently initiated empirically in hospitalized patients. Reports indicate that a majority of AECOPD admissions receive antibiotics, even when clear bacterial evidence is lacking [4]. Clinical indicators such as sputum purulence and symptom severity are commonly used to guide therapy; however, these criteria are subjective and ay not reliably distinguish bacterial from non-bacterial exacerbations [5]. This practice contributes to antibiotic overuse, antimicrobial resistance, drug-related adverse effects and unnecessary healthcare costs [6]. C-reactive protein (CRP) is an acute-phase reactant synthesized by hepatocytes in response to systemic inflammatory cytokines [7]. Elevated CRP levels are observed in bacterial infections and have been correlated with exacerbation severity in COPD. Several studies have suggested that specific CRP thresholds may help identify patients who benefit from antibiotic therapy, yet findings remain inconsistent [8]. Additionally, the role of CRP as a prognostic marker reflecting clinical recovery and short-term outcomes is not fully established. Therefore, it is of interest to evaluate CRP as both a guide for antibiotic therapy and a prognostic marker in patients hospitalized with acute infective exacerbation of COPD.

## Materials and Methods:

This prospective observational study was conducted in the Department of Pulmonary Medicine at Christian Medical College and Hospital after institutional ethical approval. Fifty consecutive patients admitted with acute infective exacerbation of COPD were enrolled. All participants fulfilled GOLD diagnostic criteria for COPD and presented with acute worsening of respiratory symptoms requiring hospitalization. Inclusion criteria comprised age ≥40 years, established severe COPD, history of smoking exceeding 10 years, at least two exacerbations annually and clinical features suggestive of infective exacerbation. Patients with cystic fibrosis, active tuberculosis, immunocompromised states, pregnancy, suspected pneumonia, prior systemic corticosteroid use for the current episode, or unwillingness to consent were excluded to minimize confounding factors. Baseline data included demographic characteristics, smoking history, comorbidities and clinical parameters. Laboratory investigations comprised complete blood count, renal and liver function tests, sputum culture, chest imaging and serum C-reactive protein levels. CRP was measured on admission (Day 1) and repeated on Days 2 and 3. Total leukocyte count was recorded concurrently. Antibiotic therapy was initiated if CRP was ≥50 mg/L. Patients with CRP <50 mg/L were managed with bronchodilators, systemic corticosteroids, oxygen therapy and supportive care, unless clinical deterioration necessitated antibiotic initiation. Clinical parameters including respiratory rate, temperature, oxygen saturation, oxygen requirement and modified Medical Research Council (mMRC) dyspnoea grade were assessed daily. Primary outcomes included need for antibiotics, clinical improvement, duration of hospitalization, ICU admission, mortality and readmission. Statistical analysis was performed using SPSS software. Continuous variables were expressed as mean ± standard deviation and compared using appropriate parametric tests. Categorical variables were analyzed using chi-square test. A p-value <0.05 was considered statistically significant.

## Results:

Fifty male patients with acute infective exacerbation of COPD were enrolled and completed in-hospital follow-up. The majority were aged between 51 and 70 years, with substantial cumulative smoking exposure. Comorbidities were common, particularly hypertension (70%) and diabetes mellitus (52%), reflecting high cardiometabolic burden in severe COPD admissions. Based on CRP-guided criteria, antibiotics were initiated in 33 patients (66%), while 17 patients (34%) were managed without antibiotics. Baseline CRP levels were significantly higher in the antibiotic group compared to the non-antibiotic group. Mean CRP declined progressively over three days in both groups, with a steeper initial reduction observed in patients receiving antibiotics. Intergroup differences in CRP were significant on Days 1 and 2 but converged by Day 3. Total leukocyte count followed a similar pattern, with higher baseline values in the antibiotic group and significant reduction over time in both groups. Respiratory rate improved steadily across hospitalization, with no statistically significant differences between groups at any time point. Baseline temperature and oxygen saturation were significantly worse in the antibiotic group on Day 1, indicating greater initial severity; however, these differences resolved by Days 2 and 3. Oxygen requirements were highest on admission, with several patients requiring high-flow oxygen or non-invasive ventilation. By Day 3, most patients were stabilized on low-flow oxygen or room air. All patients presented with severe dyspnoea (mMRC Grade 4) at admission, with rapid symptomatic improvement observed by Day 3 regardless of antibiotic administration. No increase in adverse outcomes, ICU transfer, or early deterioration was observed in patients managed without antibiotics. These findings indicate that patients with lower baseline CRP improved clinically without antibiotic therapy, while elevated CRP identified individuals with greater initial inflammatory and clinical severity. Table 1 (see PDF) shows that 96% of patients were older than 50 years, with the largest proportion in the 51-60 year (34%) and >70 year (32%) categories, indicating predominance of older adults. The cohort was entirely male and 84% had more than 10 pack-years of smoking exposure, including 38% exceeding 20 pack-years. Hypertension was present in 70% and diabetes mellitus in 52% of patients, while 14% had pulmonary tuberculosis sequelae, reflecting substantial comorbidity burden. Table 2 (see PDF) indicates that 66% of patients received antibiotics based on CRP ≥50 mg/L, while 34% were managed without antibiotics. Purulent sputum was present in 90% of cases across both groups, demonstrating that sputum character alone did not exclusively determine antibiotic administration. Table 3 (see PDF) demonstrates that baseline CRP was markedly higher in the antibiotic group (74.93 mg/L) compared to the non-antibiotic group (40.29 mg/L), with significant reduction by Day 3 in both groups. Total leukocyte count followed a similar pattern, showing higher initial values in the antibiotic group and convergence by Day 3. Table 4 (see PDF) compares clinical parameters between groups and shows significantly higher baseline temperature and lower oxygen saturation in the antibiotic group on Day 1, while respiratory rate did not differ significantly at any time point. These intergroup differences resolved by Day 3. Table 5 (see PDF) depicts a progressive reduction in oxygen requirement, with high-flow oxygen and NIV required on Day 1, shifting to predominantly low-flow oxygen or room air by Day 3. Table 6 (see PDF) highlights rapid improvement in dyspnoea severity, with all patients presenting with mMRC Grade 4 at admission and most improving to Grades 1-2 by Day 3.

## Discussion:

This prospective study evaluated CRP-guided antibiotic therapy in hospitalized patients with acute infective exacerbation of COPD and demonstrated that antibiotic initiation based on CRP ≥50 mg/L was associated with greater initial inflammatory severity but similar short-term clinical recovery compared to patients managed without antibiotics. Patients with lower baseline CRP improved without antibiotic exposure and did not show adverse clinical outcomes during hospitalization. These findings support the role of CRP as a practical biomarker for distinguishing patients who may not require empirical antibiotics [9]. Baseline CRP and total leukocyte counts were significantly higher in the antibiotic group, reflecting greater systemic inflammatory burden at presentation [10]. Both markers declined substantially over three days, indicating resolution of inflammation with standard management and where indicated, antibiotic therapy. Importantly, convergence of CRP and leukocyte levels by Day 3 suggests that patients with lower inflammatory indices may recover without antimicrobial escalation [11]. Clinical parameters including respiratory rate, oxygen saturation, oxygen requirement and dyspnoea grade improved consistently in both groups. Although the antibiotic group demonstrated more severe initial physiological derangement, intergroup differences resolved during hospitalization. This pattern indicates that CRP stratification identified patients with higher initial severity rather than determining divergent recovery trajectories [12]. The high prevalence of sputum purulence across both groups underscores the limitation of relying solely on subjective clinical indicators for antibiotic decisions. CRP provided an objective inflammatory threshold that reduced unnecessary antibiotic administration in one-third of patients without compromising clinical stability. This supports CRP as a feasible tool for antibiotic stewardship in AECOPD [13]. The study also suggests that CRP has short-term prognostic value, as higher baseline levels were associated with greater physiological instability at admission. However, rapid decline in CRP paralleled clinical improvement, reinforcing its dynamic utility in monitoring response [14]. Limitations include small sample size, single-center design and absence of long-term follow-up outcomes. Larger randomized controlled trials are required to validate CRP thresholds and evaluate long-term safety and cost-effectiveness. Overall, CRP-guided antibiotic therapy appears to reduce unnecessary antibiotic exposure while maintaining clinical safety, supporting its integration into evidence-based management of acute infective exacerbation of COPD.

## Conclusion:

C-reactive protein-guided antibiotic therapy in acute infective exacerbation of COPD effectively stratified patients based on inflammatory severity and reduced unnecessary antibiotic exposure. Patients with CRP <50 mg/L demonstrated clinical improvement without antibiotics, while elevated CRP identified those with greater initial physiological instability. Thus, CRP measurement provides a practical and objective tool to support antibiotic stewardship and improve therapeutic decision-making in hospitalized AECOPD.
